# Postdocs as Key to Faculty Diversity: A Structured and Collaborative Approach for Research Universities

**DOI:** 10.3389/fpsyg.2021.759263

**Published:** 2022-04-25

**Authors:** Colette Patt, Andrew Eppig, Mark A. Richards

**Affiliations:** ^1^Division of Mathematical and Physical Sciences, College of Letters and Science, University of California, Berkeley, Berkeley, CA, United States; ^2^Office of Equity and Inclusion, University of California, Berkeley, Berkeley, CA, United States; ^3^Department of Earth and Space Sciences, College of the Environment, University of Washington, Seattle, WA, United States

**Keywords:** postdoctoral, faculty, equity, doctoral, underrepresented minority, URM, diversity, STEM

## Abstract

Over the past 50 years the diversity of higher education faculty in the mathematical, physical, computer, and engineering sciences (MPCES) has advanced very little at 4-year universities in the United States. This is despite laws and policies such as affirmative action, interventions by universities, and enormous financial investment by federal agencies to diversify science, technology, mathematics, and engineering (STEM) career pathways into academia. Data comparing the fraction of underrepresented minority (URM) postdoctoral scholars to the fraction of faculty at these institutions offer a straightforward empirical explanation for this state of affairs. URM postdoc appointments lag significantly behind progress in terms of both undergraduate and Ph.D.-level STEM student populations. Indeed, URM postdoc appointments lag well-behind faculty diversity itself in the MPCES fields, most of which draw their faculty heavily from the postdoctoral ranks, particularly at research-intensive (R1) universities. Thus, a sea-change in how postdocs are recruited, how their careers are developed, and how they are identified as potential faculty is required in order to diversify the nation’s faculty, and particularly the R1 MPCES professoriate. Our research shows that both Ph.D. students and postdocs benefit from intentional structure at various levels of their respective “apprentice” experiences, a factor that we believe has been neglected. Several key structural approaches are highly effective in these regards: (1) A collaborative approach in which leading research universities collectively identify outstanding URM candidates; (2) Faculty engagement in recruiting and supporting these postdocs; (3) Inter-institutional exchange programs to heighten the visibility and broaden the professional experiences of these postdocs; (4) Community-building activities that create a sense of belonging and encourage continuing in academia for each cohort; and (5) Continuing research based on outcomes and new experimental approaches. The California Alliance, consisting of UC Berkeley, UCLA, Caltech, and Stanford, has been engaged in such a program for almost a decade now, with most of the California Alliance URM postdocs now in tenure track positions or on the path toward careers as faculty at research intensive (R1) institutions. If this approach was brought to scale by involving the top 25 or so URM Ph.D.-producing R1 institutions in the MPCES fields, about 40% of the national URM postdoctoral population in these fields could be affected. Although this impact would fall short of bringing URM MPCES faculty ranks up to full representation of the United States population as a whole, it would vastly improve the outlook for URM students and their aspirations to take on leadership roles as scientists and engineers.

## Introduction

Ethnic or racial minorities now constitute more than half of the United States population under age eighteen (U.S. Census Bureau, 2019; NCES, 2021). Yet, most United States scientists and engineers -- majority and underrepresented minority (URM)^1^ – will enter their professional lives without ever having a URM K-12 science teacher, university professor, or even graduate teaching assistant (Towns, 2010; Jones, 2018). Most may have no more than one or two URM science colleagues throughout their careers. While it is important to address the lack of diversity in science, technology, mathematics, and engineering (STEM) at every level, if 4-year universities, including research-intensive (R1) universities, in the United States diversify their STEM faculty, that will have a major impact that can cascade across all levels.

**FIGURE 1:**
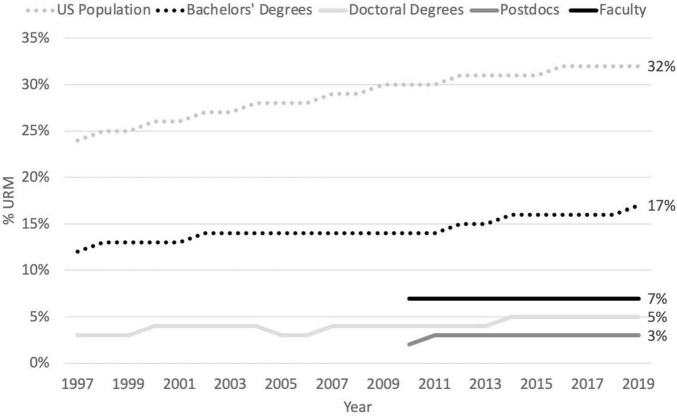
Trends in URM representation in MPCES, 2010–2019. URM (Hispanic; Black or African American, non-Hispanic; American Indian or Alaska Native, non-Hispanic; and where the data permits Native Hawaiian or Other Pacific Islander, non-Hispanic) representation in the United States has increased from 30% in 2010 to 32% in 2019. Over that same period the general population trends have been mirrored by URM representation among all MPCES bachelor degrees (including Temporary visa holders) which has increased from 14% to 17%. URM representation among MPCES doctoral degrees (including Temporary visa holders), and among MPCES postdocs (including Temporary visa holders), has increased only slightly from 4 to 5%, and 2 to 3%, respectively. URM representation among MPCES faculty has held steady at 7% over this period (CDC, 2021; NCES, 2021a,b,c).

At our nation’s 4-year universities, underrepresented minorities constitute 7% of the mathematical, physical, computer, and engineering sciences (MPCES) tenure and tenure-track faculty. This severe underrepresentation among faculty has persisted for decades, so that we have actually lost ground relative to our country’s increasing URM population (see Figure 1). In turn, the lack of URM faculty role models is discouraging to a large fraction of the United States population who could be joining and contributing to our scientific and engineering workforce (Stockard et al., 2021). Indeed, only about one-third of URM undergraduate students entering our research universities intending to major in MPCES fields persist to obtain these degrees, compared to a completion rate of approximately two-thirds by majority male students (Hsu et al., 2008; HERI, 2010; ACT, 2013; Chen, 2013; Wadhwani and Eppig, 2018; NSF, 2019).

### Why So Little Progress?

Despite the passage of Title VII, which barred discrimination on the basis of race and gender in higher education employment, for decades the diversity of the STEM faculty did not increase beyond tokenism. Affirmative action policies also did not fundamentally alter the demographics of the STEM faculty (Wood et al., 2008). Then, in 1996, Proposition 209 passed in California, banning affirmative action in California, and similar laws passed in other states. The elimination of the National Science Foundation’s (NSF) Minority Graduate Research Fellowship program during this period, in 1998, exemplifies the way that political winds were reframing how diversity could be addressed in higher education (Muller-Parker et al., 2020). Universities and federal agencies sought new approaches to diversify STEM (Malcom, 1976; Duderstadt, 2015; Phillips, 2019).

Universities and federal agencies began to focus keenly on diversifying the undergraduate and graduate ranks of STEM students through outreach and recruitment of “diversity” students into STEM. At the faculty level, the ADVANCE program focused on institutional change to improve conditions for women faculty, but did not address the postdoctoral level. The federal government’s science agencies also invested in this effort (Dero et al., 2019). For example, the Government Accountability Office reports that in 2016 approximately $2.9 billion was spent on STEM education and diversity programs, of which the NSF received $1.2 billion (Clark and Esters, 2018; GAO, 2018).

The NSF progressed from its focus, starting in 1991, on undergraduates through the Louis Stokes Alliances for Minorities Program (LSAMP), to graduate recruitment in its Minority Graduate Education (MGE) program, starting in 1998, then graduate retention in the MGE program, which was renamed the Alliances for Graduate Education and the Professoriate (AGEP), and recently, has widened its focus through the AGEP program to include models that address diversity at the postdoc and faculty levels. The newest NSF diversity program, started in 2016, is INCLUDES, which supports linkages across educational levels and institutional types to increase diversity—but also excludes a distinct and substantial focus on the postdoctoral level. This progression over the past 30 years exemplifies the excruciating slowness of recognition at either federal grant-making agencies or universities that diversifying the faculty will take more than increasing URMs in the bachelor’s degree (BA) or doctoral degree (Ph.D.) pools. While the NSF’s focus on the undergraduate and graduate educational years certainly is necessary, it has been insufficient for increasing the available pool of candidates to enter the faculty.

Turning to university efforts, one of the main foci of advocates for diversifying the faculty has been addressing bias in faculty searches and hiring processes. A plethora of guidelines, training materials and requirements, and an accompanying growth in diversity specialists and consultants has emerged to guide search committees and department leaders. Much of this push for change relies on teaching the members of search committees about psychological findings on how bias enters into decision making, inclusion of women and minorities on search committees, active outreach, and requirements that candidates offer their views in “diversity statements” (Goulden et al., 2019; UCOP, 2019). This approach is valuable in alerting search committees to considerations for equitable hiring when there is a diverse pool of applicants, encouraging search committees to engage in active outreach (Clauset et al., 2015), and signaling that diversity matters to the department and institution, but it too has been insufficient.

These important thrusts toward diversification of the STEM faculty have ignored the final turning point on the path to the professoriate: the postdoctoral experience. Completing a postdoctoral experience was once an expectation for prospective faculty only in a limited set of STEM fields, but over the past two decades, this requirement has expanded across STEM fields rapidly, and in some fields, escalated into an expectation that competitive candidates will complete long or multiple postdoctoral fellowships. At doctoral granting research universities, in particular, which are the largest employers of tenure-track faculty (AAUP, 2018), a Ph.D. is rarely sufficient for winning a MPCES faculty job – most faculty are recruited from the postdoctoral ranks (AAU, 1998; Su, 2013; Yang and Webber, 2015). Indeed, most scientific and increasingly most engineering professional positions in STEM research, are no longer filled by new Ph.D. recipients, but rather by postdoctoral researchers. This is true not only with respect to faculty at research universities, but also for research scientists at Federally Funded R&D Centers (FFRDCs), and in research and development (R&D) in private industry.

Yet despite this reality, the “URM availability pool” for faculty hiring continues to be defined as the number and percentage of URM Ph.D.s, with university administrators unaware of or not recognizing the expanded credentialing that faculty now require as they assess junior colleagues’ candidacy for tenure track positions (Stacy et al., 2018; University of Michigan, 2018; Cornell University, 2021). This reliance on the demographics of the graduating cohort of Ph.D.s, rather than the demographics of the cohort of employed postdocs, to define URM availability pools for faculty jobs is a widespread “blindspot” that obfuscates the challenge of diversifying the faculty. It should therefore come as no surprise that little progress has been made in diversifying the professoriate, or that the problem is acute at research universities.

As a result of the last two decades of inaction to diversify the postdoctoral level, the fraction of URM Ph.D. degree recipients in the MPCES fields has increased from about 4% to about 5%, but, shockingly, the fraction of URM postdoctoral scholars has remained even smaller, increasing only from about 2% to 3% of all postdocs, including foreign nationals (see Figure 1; U.S. Department of Education, 2010–2019a,b,c; CDC, 2021). Among United States citizens and residents, the fraction of URM Ph.D. recipients in MPCES has increased from 9% to 11%, and URM postdocs from 6% to 7%.

Diversifying the postdoctoral level is complicated by several factors, especially the highly decentralized sources of postdoctoral fellowships, the atomized locations of postdoctoral scholars, and the short duration of these positions. Most postdoctoral fellowships in academia attach to extramural grants won by individual faculty, who, as principal investigators (PIs), select and hire postdocs, often seeking candidates with niche technical training best suited to the focus of their grants. When grants are made, they tend to be of relatively short duration and the need to hire quickly, therefore, is pressing to a PI if they are to yield results during the award period. In this context, it is understandable that PIs turn to their own scientific networks, perhaps their own advisers or former students, to identify qualified individuals, and that they, usually alone, hire the postdoc of their choice.

Though this conventional approach to postdoc hiring makes sense in context, it is, in practice, a closed system, easily taking on the qualities of a proverbial “old boys’ network.” Universities tend not to impose requirements for advertising these positions, perhaps for pragmatic reasons, and indeed, there tend to be few uniformities in postdoctoral fellowship hiring across institutions, or even within institutions. In some contexts, postdoctoral fellowships are understood to be the direct route into a permanent position, thereby extending the problem with this closed system of hiring postdocs to the next professional level. Given the way postdoctoral fellows are hired and the reality that their professional lives often are experienced in a single lab with a direct report to the person who hired them, it is rare for mid-level or high-level administrators to recognize the cumulative demographics of a department’s or other campus unit’s postdoctoral population.

From a graduating student’s perspective, the main approach to finding postdocs usually involves a somewhat random walk through unlinked websites of postdoc programs, dependence on *ad hoc* scientific networks, and the attentiveness of their Ph.D. advisers. Once in a postdoc, this kind of *ad hoc* process for career advancement can worsen, with increased isolation and uncertainty. Often postdoc scholars’ network expansion – critical for advancement to the ranks of the faculty or professional research positions that lead to leadership in the scientific community – becomes almost entirely dependent on a single postdoc mentor and a postdoc’s own initiative. The prospects of one’s fate being sealed by a career step that is crucial for scholarly and career advancement, but difficult to win and with uncertain outcomes, and that is generally experienced in a new geographic location without scientific, institutional, or familiar community supports, can be daunting for graduating students (Ferguson et al., 2017). For those without financial safety nets, the uncertainties of the postdoc stage may seem too risky (Ferguson et al., 2017). Many turn away at this stage.

Exacerbating the problem is the reality that it would be exceptional for any institution to consider the diversification of the postdoc level (or even their own postdoc population) to be a high-level priority, even those with deep commitments to diversity at every other educational and career level. Most universities have little incentive to increase postdoc diversity, relative to their incentive to increase graduate student or faculty diversity. In part this is a result of the national inattention to the postdoctoral level, in general. This inattention is illustrated by the key recommendation of the National Postdoctoral Association in its most recent report. It calls for the provision of adequate institutional resources to staff institutional postdoc affairs offices, and to achieve equality in benefits, offer adequate parental leave and family-friendly policies, and track postdocs after they leave the institution (Ferguson et al., 2021). And so the problem persists — not only unaddressed, but also largely unrecognized.

### What to Do?

First, universities and federal funding agencies must recognize the problem. The continued homogeneity of the postdoc pool makes diversifying the nation’s MPCES faculty an intractable problem. Secondly, the scientific community, universities, and federal agencies must acknowledge the complexity of diversifying the postdoctoral population, a challenge that is not akin to diversifying the educational experiences that precede it, nor the professional positions that proceed from it. To address this problem, the scientific community must identify outstanding URM Ph.D. candidates, encourage promising URM graduate students to pursue postdocs at research universities, increase their awareness of available postdoctoral jobs and the awareness of their scientific accomplishments among those who can hire them as postdocs, approach both postdoc hiring and career advancement beyond the postdoc with intentionality and coordination, support URM postdocs in their ambitions to successfully seek faculty positions, increase the visibility of URM postdocs among those who are in positions to hire them as faculty, and ensure that both for postdoctoral and faculty positions, advertising, selection, and hiring processes are free of bias.

### A Call for Leadership

Addressing underrepresentation at the postdoctoral level requires a coordinated national effort that goes beyond local programs or initiatives, and requires new leadership from granting agencies (especially NSF), professional societies, and research universities.

Most efforts to date rely upon parallel but separate tracks of funding to recruit URM postdocs and postdocs who, in other ways, contribute to diversity. Exemplars of these approaches are the University of California’s President’s Postdoctoral Fellowship Program and its partner programs, as well as the new AGEP Promise Academy Alliance. These are immensely valuable programs in offering opportunities for scholars who will contribute to faculty diversity to advance within these institutions, but they are not designed to, and cannot address the problem of underrepresentation at the national scale.

Instead, we argue for a strategy that connects graduate students to prospective mentors nationally with far greater intentionality and inter-institutional cooperation – a common applicant pool, mentored inter-institutional visits, multi-layered professional development, a “concierge” approach to linking highly sought-after URM advanced graduate students to prospective postdoc mentors, and national and institutional recognition of the importance of focusing resources and attention on diversifying the postdoctoral level. This approach would diversify the MPCES professoriate by leveraging existing structures and norms to mainstream the success of URM postdocs as faculty candidates. Given that the fraction of URM MPCES Ph.D.s is currently almost double that of postdocs and is steadily (albeit far too slowly) increasing, truly significant change should be possible within just a few years.

### A Solvable Problem

The time has come to work with common purpose, and at scale, to generate a diverse professional scientific community. Beyond focusing on undergraduates, graduate students, and faculty, we must address a key overlooked population—postdoctoral scholars.

## A Wake-Up Call: Publication and Structure

### Laying a Foundation

For over a decade the authors have convened a STEM Diversity Research Group at the University of California, Berkeley, consisting of the Dean for Mathematical and Physical Sciences, prominent diversity program directors, faculty and graduate students in psychology and sociology, and institutional data analysts. Initially funded by the Mitchell Kapor Foundation (now the Kapor Center for Social Impact) and the National Science Foundation, our group dutifully undertook an intensive survey of both graduate and undergraduate students in the mathematical, physical, and computer sciences in order assess various aspects of student life in the STEM fields at Berkeley, and to lay groundwork for addressing racial, ethnic, and gender disparities.

The Berkeley Life in Science Survey (BLISS), conducted in 2013–2014, consisted of many of the standard questions regarding progress to degree, mentorship, financial support, etc. However, the survey also queried graduate students as to whether they had participated as an author on a paper submitted for publication in the past year. As it turns out, there were almost no previous studies regarding this issue. However, results from this question opened an entirely new avenue for research, and provided important insights for future progress in STEM diversity.

### Publication Disparities

**FIGURE 2:**
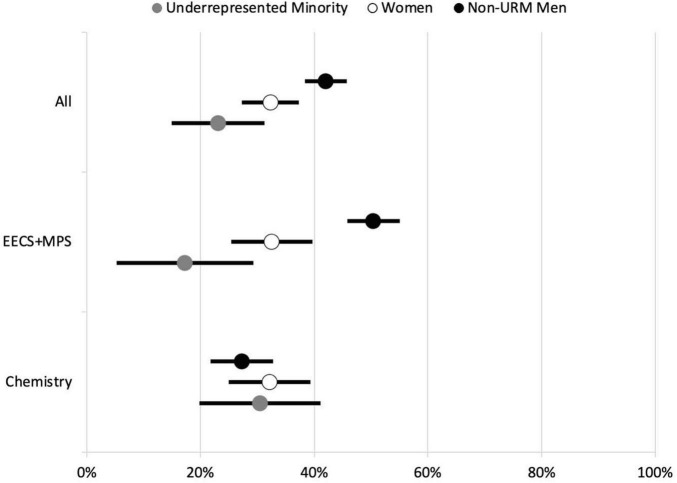
Berkeley doctoral student paper submission rates in the mathematical, physical, and computer sciences. Self-reported paper submission rates differ greatly among URMs (African American, Chicanx/Latinx, and Native American/Alaska Native), women, and non-URM men for Berkeley Ph.D. students in the mathematical, physical, and computer sciences with non-URM men having the highest rates of papers submitted for publication (42%) followed by women (32%) and then URMs (23%). These differences are exacerbated in Electrical Engineering and Computer Science (EECS) and Mathematical and Physical Sciences (MPS) but disappear in Chemistry. Error bars show 95th percentile confidence intervals. Figure adapted from Mendoza-Denton et al. (2017).

The results of this work have been published in detail elsewhere (Mendoza-Denton et al., 2017), but the most important outcome is summarized in Figure 2. When we aggregated all Ph.D. student respondents, we found that both underrepresented minority men and women (URM) and non-URM women students were significantly less likely to have submitted a paper for publication in the last year than their male non-URM (white and Asian-American) counterparts – URM’s were only about half as likely to have submitted a paper for publication, which was quite disturbing, but also suggested a clue to explaining disparate career outcomes for Ph.D. students.

As discussed by Mendoza-Denton et al. (2017), these results were carefully controlled for such confounding variables as number of years in the Ph.D. program, advancement to candidacy, and time spent employed in research, teaching and on fellowship, but the results of Figure 2 remained robust.

We quickly sought to understand if these results were department-specific, and in that process one result stood out in stark relief. As indicated in Figure 2, Ph.D. students in Berkeley’s College of Chemistry did not show resolvable disparities in publication rates according to race/ethnicity or gender, whereas the remainder of the survey group consisting of the Departments of Astronomy, Earth and Planetary Science, Mathematics, Physics, Statistics, and Electrical Engineering and Computer Science showed even greater disparities with the Chemistry respondents separated out.

Fortunately, we quickly found that an independent survey conducted by Berkeley’s Graduate Division offered unequivocal support for these findings. This survey had been administered from 1998 to 2015, was completed by 98% of all graduating Ph.D. students at Berkeley, and included questions about publication similar to those in the BLISS survey. As described in Mendoza-Denton et al. (2017), the Graduate Division survey yielded essentially the same results with many more participants and much greater longitudinal control: as a whole URM Ph.D.’s in the MPCS fields at Berkeley were only about half as likely to publish as their male non-URM counterparts, again with the conspicuous exception of Chemistry, where publishing frequency was statistically independent of race/ethnicity and gender. Clearly, Berkeley’s College of Chemistry had figured out something about eliminating disparities that other departments had not!

### What’s So Special About Berkeley Chemistry?

To gain insight, we conducted qualitative research. Berkeley is unique in that the Departments of Chemistry and Chemical Engineering, both consistently ranked as top departments nationally, constitute an independent College of Chemistry, with an equally unique culture for graduate study. In these programs, students entering the College of Chemistry encounter a highly structured environment in which expectations for selection of advisers, the timeline for conducting research, writing, and publication are made clear at the outset. Most of the features of this structured approach pertain to progress through the first few years of the program, establishing both short-and long-term expectations and creating the conditions under which these expectations can be met, with hands-on involvement and management of each stage of the process by designated faculty who are not the students’ research advisers. The following practices exemplify the highly structured process for Chemistry Ph.D. students: students are expected to begin writing their first paper no later than their second year—they are required to submit a formal paper or proposal on which they receive comments from two faculty; there is a routinized approach, managed by a designated faculty member, to the matching of students and thesis advisers; students are required to meet with and rank their choices of advisers, and faculty to rank their choices of students, thus enabling multiple advisors to become aware of student progress at an early stage of the graduate program.

It is also noteworthy that the Berkeley Chemistry doctoral program has been heralded as the most successful in the country in terms of placing it’s women Ph.D.s into faculty positions at doctoral (R1) universities (Laursen and Weston, 2014).

### Why Does All This Matter?

For many years, studies of disparate outcomes in STEM have focused on recruitment (who gets admitted to elite Ph.D. programs?) and normative outcomes such as advancement to candidacy and degree completion, as well as mentoring relationships and financial support, and of course both implicit and explicit bias. All of these factors matter, but even mitigating for such factors it is widely understood that the single most important factor that influences whether a newly graduated Ph.D. or postdoctoral scholar makes the short list or is hired for a faculty position in a research university is their publication record (Van Dijk et al., 2014; Fernandes et al., 2020).

To put it bluntly, if URM Ph.D.’s publish only half as much as non-URM’s, they are at a serious disadvantage in highly competitive searches. Thus, we need to know what factors contribute to equitable outcomes in terms of publication of research results for graduate students.

### Structure and Belonging

From our work we have come to advocate for a new paradigm in which the more traditional notions of mentorship, community, and sense of belonging are complemented by the equally important notion of structure, wherein the norms and expectations for advanced study in STEM are made clear to all. URM students often do not arrive in graduate programs with the same amount of social or cultural capital that is valued in academia as their non-URM counterparts –in part because they are more likely to be first-generation college students from relatively low-income economic backgrounds, and therefore are less likely than their peers to have attended elite high schools and colleges, and are less likely to have grown up in close contact with professionals or academics.

At the graduate level, the research literature shows that graduate and postdoctoral education for URM students, fails to provide key experiences. Chief among these is a sense of belonging in the community (Mendoza-Denton et al., 2002; Walton and Cohen, 2007, 2011; Good et al., 2012). A lack of belonging often arises from being excluded, intentionally or otherwise, from the informal social networks and gatherings where critical information and budding collaborations occur (Austin, 2002; Nettles and Millett, 2006). Importantly, URM students are systematically provided with poorer mentorship relative to their majority group peers, either because of bias (Moss-Racusin et al., 2012) or apprehension around intergroup mentorship (Mendoza-Denton et al., 2002, 2018a; Crosby and Monin, 2007; Mendoza-Denton and Page-Gould, 2008; Page-Gould et al., 2010). There is reason to believe that postdoctoral scholars may experience isolation and stress even more acutely than graduate students (Arnold, 2014). Thus, a robust literature documents systematic limitations in STEM education around networking, information sharing, belonging, and community for URM scholars (Walker et al., 2008). In our previous research, we have noted that clarity of expectations and a sense of belonging are critical aspects of programs that aim to achieve equity in outcomes (Mendoza-Denton et al., 2017; Mendoza-Denton et al., 2018b; Fisher et al., 2019). For all these reasons, URM students may only realize the key importance of publication (as opposed to degree completion) relatively late in their graduate studies unless such expectations are made explicit at the outset. The “secret handshake” culture of many academic fields does not always work well for URM students.

Indeed, the research demonstrates more generally the simple principle that “ambiguity is the breeding ground for bias” (Mendoza-Denton et al., 2018b). But clearly the Chemistry doctoral program at Berkeley has short-circuited this source of bias in ways that have yielded equitable outcomes in a fashion that we consider spectacular relative to most STEM Ph.D. programs at R1 universities in the United States (Laursen and Weston, 2014; Fisher et al., 2019).

## California Alliance Formation and Program Design

### Structural Bias and Systemic Inertia

The results summarized in the foregoing section could be characterized as a particular, and in our opinion particularly important, form of structural bias. In fact, lack of structure, or ambiguity, regarding norms and expectations in many if not most STEM Ph.D. programs is what we have highlighted above. Lack of publications is but one symptom of this kind of bias, and in this section, we address a broader interventional approach that includes addressing structural bias that works to disadvantage both Ph.D. students and postdoctoral scholars, especially when it comes to pursuing, or even envisioning, careers as faculty at leading research universities.

Over the past decade we have focused on diversifying the populations of both advanced Ph.D. students and postdocs in the MPCES fields for reasons made clear in the opening section of this paper —unless these populations become much more diverse there is no way that the professoriate will do so. In order to explain our approach, we begin with some observations:

(1) Most postdocs have traditionally been recruited through back-channel means, typically one professor recommending a finishing Ph.D. student to a professor at another institution. True searches for postdoc positions remain rare. This constitutes the ultimate “old boy network,” in which mostly white male professors recommend their mostly white and Asian male students to other mostly white male professors.
(2) It is rarely a high priority for individual institutions to pay much attention to the diversity of their postdocs, even if they are keen on diversifying their own graduate student and professorial ranks, because postdocs most commonly do not remain at their host institution for faculty positions.
(3) Federal agencies have until only very recently paid little attention to the diversity of the postdoc ranks, as these positions are more difficult to track than graduate students, and norms for mentoring and support are highly variable.
(4) Expectations for the postdoctoral experience remain poorly defined; as a result, postdocs often are in a kind of limbo state in most research groups, with few assurances of specific normative outcomes (e.g., degrees) other than the next job, academic or otherwise.
(5) though postdocs are widely understood to be a rite of passage for most MPCES faculty positions at R1 universities, the social capital gap for URM Ph.D. students described in the previous section can become exacerbated by a lack of clarity as to how this step actually works in practice, and compounded by uncertainty of the career outcome, particularly for scientists from low-income backgrounds.

### California Alliance Inception, Design, and Outcomes

For the above reasons, four leading research universities in California – Berkeley, Stanford, UCLA, and Caltech – undertook in 2011 to band together to build upon their collective prestige and interest in diversifying their Ph.D. student, postdoctoral, and professorial ranks. Thus was born the California Alliance. The principal motivating factor behind this unprecedented grouping was to overcome structural bias through collaboration to identify and nurture the careers of aspiring URM MPCES scholars by introducing new practices to their recruitment and development. Out of many creative and fruitful discussions among academic leaders at the four California Alliance institutions eventually sprang the following parallel approaches that have combined to yield great success. The California Alliance’s collaborative efforts lead to the hiring of 40 URM postdocs across the four universities.

(1) Initially, key to the Alliance’s approach was a national solicitation on the part of all four institutions to identify outstanding URM candidates for postdoctoral appointments across the MPCES fields, but with a new and key ingredient —all the applicants for the California Alliance postdocs could be considered and recruited by any, or all, of the four institutions. The solicitation was distributed widely to scientific organizations and associations of URM scientists, contacts of the California Alliance partners’ faculty, and directors of program serving URM. This made the solicitation very attractive nationally, resulting in more than 60 applicants most years. NSF funding provided for approximately five postdoctoral fellowships over approximately 6–8 years, but it was understood among the four partner institutions that they needed to come up with significant matching funds, so that the NSF resources could be stretched, and more outstanding URM candidates hired. The final institutional/NSF matching ratio turned out to be more than 7:1. Put another way, once an exciting candidate pool was developed, the partner institutions were eager to hire the applicants. This constituted an interruption of the traditional, proverbial “old boy network” for postdoctoral hiring, and brought the Alliance successful candidates whom they otherwise would never have known about.
(2) In time, with a growing cadre of both advanced URM Ph.D. students and postdocs within the Alliance, the member institutions decided to further leverage their collective prestige to further interrupt systemic structural bias. This resulted in the formation of the Research Exchange, wherein advanced Ph.D. students and postdocs were invited to experience 1- to 2-week mentored visits with research groups at the other participating institutions in order to expand their scientific experience and horizons at critical career stages, and to increase their visibility as potential faculty members. This approach involved minimal costs (mainly travel), and has turned out to be both extremely effective and very popular, both with visiting candidates and their respective inter-institutional mentors.
(3) The third structural element of the California Alliance can be thought of as “career development,” with annual retreats and informal networking among participants being the most prominent activities. Each year one institution hosted a 2- to 3-day pan-Alliance retreat for students, postdocs, and faculty across all four institutions. Activities included brief scientific presentations/posters, breakout sessions on mentoring, publication, applying for jobs, addressing bias, etc., and social activities to form relationships and increase comfort and familiarity with the professorial world. In fact, these retreats proved to be just as popular among faculty as students and postdocs, with many faculty who had never before participated in diversity work becoming inspired by and heavily involved with the diversity goals of the Alliance.
(4) The California Alliance partners also worked together on applied social science research focused on better understanding and addressing the reasons for continuing underrepresentation of minorities at the advanced levels of the scientific community (e.g., Fisher et al., 2019).

Recently, the Alliance has expanded (with new NSF support) to include five other leading R1 universities – University of Michigan, The University of Texas at Austin, University of Washington, Georgia Institute of Technology, and Harvard University. Most of the above program elements remain active in this new Research Universities Alliance (RUA), which we hope will lead to a larger national effort and greater national impact.

## Putting the California Alliance Postdocs in a National Context

The California Alliance started hiring postdocs in 2015, and as of 2019 it employed an average of 19 underrepresented minority (URM) postdocs per year^2^ (Table 1). In the 5 years prior, Alliance institutions employed an average of 38 URM postdocs per year in MPCES^3^ which represented 1.9% of the postdocs in those fields at Alliance institutions. In the 5 years after the Alliance started hiring postdocs, Alliance institutions employed an average of 43 URM postdocs per year in MPCES fields, which represented 2.1% of postdocs in those fields at Alliance institutions ---a 15% increase in the number of URM postdocs and a 13% increase in the share of URM postdocs. Over those same periods, national URM postdocs in MPCES increased by just under 5% and declined by 1% as a share of all MPCES postdocs. If national URM postdoc share in MPCES had increased by the 13% seen by the Alliance opposed to the 1% decline actually observed, it would have translated to an increase of 68 URM postdocs in MPCES employed per year. At peer institutions^4^ (NRC, 2011) to the California Alliance in MPCES fields, URM postdocs declined by 17% in absolute numbers and declined by 21% in terms of representation among all MPCES postdocs. If the URM postdoc share in MPCES at peer institutions had increased by the 13% seen by the Alliance opposed to the 21% decline actually observed, it would have translated to an increase of 66 URM postdocs in MPCES employed by peer institutions per year^5^ (Table 1).

**TABLE 1:** National context for URM postdocs.

	Yearly averages – All		Yearly averages – URM		Shares – URM	
Group	2010–2014	2015–2019	2010–2014	2015–2019	2010–2014	2015–2019
All fields	62,893	64,867	2,595	2,878	4.1%	4.4%
Science and Engineering	44,060	45,872	1,606	1,799	3.6%	3.9%
MPCES	18,009	18,942	492	515	2.7%	2.7%
MPCES – peer institutions	6,223	6,556	183	151	2.9%	2.3%
MPCES – CA Alliance	1,978	2,019	38	43	1.9%	2.1%
CA Alliance postdocs		19		19	n/a	100.0%

*Sources: California Alliance, U.S. Census Bureau (2019), NCES (2021a,b, c), NSF Survey of Graduate Students and Postdoctorates in Science and Engineering.*

During 2015–2019, the four California Alliance institutions hired 8% of all URM postdocs in MPCES, and its twenty peer institutions hired 29% of all URM postdocs in MPCES. The California Alliance itself hired just under 4% of all URM postdocs in MPCES despite having only 0.1% of all MPCES postdocs. Ideally, the California Alliance institutions will continue to increase the number of URM postdocs in MPCES fields until the alliance represents at least 11% of all URM MPCES postdocs— as it employed 11% of overall MPCES postdocs from 2015 to 2019.

Of the 40 URM postdocs hired by the California Alliance over 2015–2019, 21 of them (53%) are currently in tenure-track faculty positions and an additional 6 (15%) are still postdocs and are still in the pool to become faculty in the future.

Previous studies have estimated the national hiring rates of postdocs to be around 15% (McConnell et al., 2018), but field and institution-specific data are not available in aggregate much less disaggregated by race/ethnicity. Given these limitations it is hard to say definitively whether the 53% hiring rate of California Alliance URM postdocs in MPCES is higher or lower than peer trends. In the future, it might be possible to use Early Career Doctorates Survey data (NCES, 2017) to estimate the relevant trends, but at present this data cannot be used for this purpose given publicly available tables.

Within the first few years of operating the Research Exchange (2017–2019), 105 advanced graduate students and postdoctoral fellows applied to participate. Of these applicants, 32 URM advanced graduate students and postdoctoral fellows completed visits to faculty, labs, and research groups of interest within the alliance institutions before the Covid-19 pandemic prevented travel. Of these 32 participants, when the California Alliance’s National Science Foundation grant ended in 2021, 11 were continuing their graduate studies, three had become faculty, 16 had continued to postdoctoral positions and two had taken positions in industry (NSF, 2022). Since then, despite complications with travel during the Covid-19 pandemic, the Research Exchange expanded as part of the Research University Alliance with an increasing number of participants subsequently taking faculty positions. The initial successes of the California Alliance’s (and now RUA’s) Research Exchange in encouraging continuation on the academic path through the advanced graduate years to the postdoc and to the faculty, along with the postdoc program’s success in advancing URM graduate students into postdocs that make them competitive for and interested in taking tenure track jobs, offer promising new approaches for strategies that can be taken to scale in the United States.

## Conclusion

Achieving racial, ethnic, and gender diversity in the STEM disciplines is a national imperative. However, over the past half-century startlingly little progress has been made, especially among faculty in United States research universities. The non-biological sciences, including the MPCES fields, have proven particularly resistant to change, which has been the focus of our work (Li and Koedel, 2017; Meyers et al., 2018). Here we have emphasized two particular aspects of the problem and solution pathways. First, in most of the MPCES fields, the lack of diversity among faculty parallels a long-neglected lack of diversity among the population of postdoctoral scholars, who are commonly recruited to fill the professorial ranks. Second, structural bias (or lack of programmatic structure) persists in both graduate programs and the postdoctoral programs they feed, and is more of a barrier than has previously been recognized.

Following on these basic observations, we have implemented a program targeted at interrupting systemic bias by developing a collaborative effort among leading research universities, focusing on both advanced Ph.D. students and postdocs in the MPCES fields. Essential elements of this program include combining institutional resources to recruit (and hire) a strong applicant pool of underrepresented minority (URM) postdoctoral candidates; inter-institutional visits by both Ph.D. students and postdocs to increase their visibility, broaden their experience, and elevate their career aspirations; professional development at all career stages leading to the professoriate, including pan-institutional retreats and extensive faculty involvement; collaborative sociological research across the consortium to test out new ideas and approaches to mitigation of historical bias.

This consortium, which now includes nine institutions, has yielded tangible results far exceeding the success of other approaches with which we are familiar. In particular, a remarkable fraction of our Ph.D.s and postdocs are successfully seeking faculty positions at R1 universities. These nine universities, together, employ 21% of the nation’s URM MPCES postdoctoral fellows. Indeed, only approximately 52 universities have track-records of hiring any MPCES URM postdoctoral fellows, according to data from the Survey of Earned Doctorates. These nine universities, together, also educate 14% of the nation’s URM Ph.D.s. This success suggests that scaling these mitigations to perhaps the top 25 or so URM Ph.D.-producing institutions in the MPCES fields would dramatically increase the fraction of URM faculty in the United States, and in turn lead to a much more robust cadre of mentors (Allen et al., 2004; Boykin et al., 2015; NASEM, 2019) for the burgeoning numbers of URM undergraduate students seeking careers in STEM.

## Data Availability Statement

Publicly available datasets were analyzed in this study. This data can be found here: U.S. Census Bureau. Population Division. “Annual Estimates of the Resident Population by Sex, Race, and Hispanic Origin for the United States: April 1, 2010 to July 1, 2019 (NC-EST2019-SR11H)” Release Date: June 2020. Based on data accessed in August 2021. U.S. Department of Education, National Center for Education Statistics, Integrated Postsecondary Education Data System (IPEDS), 2010–2019, Degrees Awarded by Colleges and Universities. Retrieved from https://ncsesdata.nsf.gov/builder/ipeds_c on August 14, 2021. U.S. Department of Education, National Center for Education Statistics, Survey of Graduate Students and Post-doctorates in Science and Engineering, 2010–2019, Post-doctorates. Retrieved from https://ncsesdata.nsf.gov/builder/gss on August 14, 2021. U.S. Department of Education, National Center for Education Statistics, Survey of Doctorate Recipients, 2010, 2013, 2015, 2017, and 2019, Table 19. Retrieved from https://www.nsf.gov/statistics/srvydoctoratework/ on August 14, 2021.

## Ethics Statement

Data presented here from research by the authors that involved human participants is cited from studies reviewed and approved by the Committee for the Protection of Human Subjects, University of California, Berkeley. The participants provided their written informed consent to participate in those studies. All other data involving participants is available from public sources.

## Author Contributions

All authors made substantial contributions to the conception and design of the work and acquisition, analysis, and interpretation of data for the work, drafted the manuscript and revised it critically for important intellectual content, provided approval for publication of the content, and agreed to be accountable for all aspects of the work in ensuring that questions related to the accuracy or integrity of any part of the work are appropriately investigated and resolved.

## Author Disclaimer

Any opinions, findings, and conclusions or recommendations expressed in this material are those of the author(s) and do not necessarily reflect the views of the National Science Foundation.

## Conflict of Interest

The authors declare that the research was conducted in the absence of any commercial or financial relationships that could be construed as a potential conflict of interest.

## Publisher’s Note

All claims expressed in this article are solely those of the authors and do not necessarily represent those of their affiliated organizations, or those of the publisher, the editors and the reviewers. Any product that may be evaluated in this article, or claim that may be made by its manufacturer, is not guaranteed or endorsed by the publisher.

## Footnotes

1 Underrepresented minority or URM is defined as African American, Chicanx/Latinx, Pacific Islander, or Native American/Alaska Native.

2 Annual counts are averaged to get postdocs employed per year.

3 MPCES is defined as Mathematics, Geosciences, Physical Sciences, Engineering, and Computer Science fields.

4 Peer institutions are defined as institutions with more than one MPCES program in the top 20 of both the statistical- and survey-based rankings from National Research Council 2011. *A Data-Based Assessment of Research-Doctorate Programs in the United States (with CD)*. Washington, DC: The National Academies Press. https://doi.org/10.17226/12994. Peer institutions, according to this definition, are: Caltech, MIT, UC Berkeley, Stanford, Harvard University, Princeton University, UC Santa Barbara, U Michigan, Northwestern, W Washington, Cornell, Georgia Tech, Carnegie Mellon, NYU, Penn State, U Arizona, UCLA, UC San Diego, U Chicago, U Colorado Boulder, UIUC, U Minnesota Twin Cities, UT Austin, U Wisconsin Madison.

5 This hypothetical is almost as large as the hypothetical for national MPCES due the much larger decline of URM postdocs at peer institutions.
