# Supplementary material for: Postdocs as Key to Faculty Diversity: A Structured and Collaborative Approach for Research Universities
Source: Front Psychol. 2022 Apr 25;12:759263. doi: 10.3389/fpsyg.2021.759263 (PMC9083322; doi:10.3389/fpsyg.2021.759263)
Supplement: Supplementary file 1 [file Table_1.pdf]

Supplementary Table 1: Trends in URM Representation in MPSCE, 2010-2019

Year	US Population	Bachelors in MPSCE	PhDs in MPSCE	Postdocs in MPSCE	Faculty in MPSCE
2019	32%	17%	5%	3%	7%
2018	32%	16%	5%	3%	n/a
2017	31%	16%	5%	3%	7%
2016	31%	16%	5%	3%	n/a
2015	31%	16%	5%	3%	7%
2014	31%	16%	5%	3%	n/a
2013	30%	15%	4%	3%	7%
2012	30%	15%	4%	3%	n/a
2011	30%	14%	4%	3%	n/a
2010	30%	14%	4%	2%	7%

Sources: Annual Estimates of the Resident Population by Sex, Race, and Hispanic Origin; IPEDS Completions Survey from Department of Education; Survey of Graduate Students and Postdoctorates in Science and Engineering; Survey of Doctorate Recipients (not administered in all years)
