# Supplementary material for: Postdocs as Key to Faculty Diversity: A Structured and Collaborative Approach for Research Universities
Source: Front Psychol. 2022 Apr 25;12:759263. doi: 10.3389/fpsyg.2021.759263 (PMC9083322; doi:10.3389/fpsyg.2021.759263)
Supplement: Supplementary file 2 [file Table_2.pdf]

Supplementary Table 2: Doctoral Student Paper Submission Rates

Group	Division	N	No	Yes	% Yes
Non-URM Men	Chemistry	66	48	18	27%
	EECS+MPS	115	57	58	50%
	All	181	105	76	42%

Group	Division	N	No	Yes	% Yes
Women	Chemistry	109	74	35	32%
	EECS+MPS	83	56	27	33%
	All	192	130	62	32%

Group	Division	N	No	Yes	% Yes
URM	Chemistry	23	16	7	30%
	EECS+MPS	29	24	5	17%
	All	52	40	12	23%

Source: Berkeley Life in Science Survey (BLISS) conducted in 2013–2014. Figure adapted from Mendoza-Denton R, Patt C, Fisher A, Eppig A, Young I, Smith A, et al. (2017) *Differences in STEM doctoral publication by ethnicity, gender and academic field at a large public research university*. PLoS ONE 12(4): e0174296. https://doi.org/10.1371/journal.pone.0174296
